# Bacteriology and the Medical Treatment of Disease

**Published:** 1908-01-04

**Authors:** 


### The Hospital

A Journal of

The Medical Sciences and Hospital Administration.

New Series. No. 42, Vol. II. [No. 1114, Vol. XLIII.] SATURDAY, JANUARY 4, 1908.

### Bacteriology and the Medical Treatment of Disease.

The study of the causes of the various diseases to which man is liable has been a conspicuous feature of medical progress during the last generation. It has come to be generally recognised that to regard some prominent symptom or physical condition as a disease, and to name and treat this without further inquiry, is a position which is no longer capable of defence. Hence has arisen the desire to get behind the superficial phenomena of every pathological disturbance to the essential causes upon which such phenomena depend. Attempts in this direction, it is felt, are not only in harmony with the claims of modern scientific method, but also afford the best prospects both of curative and of preventive treatment. To such an end an enormous and enduring impetus was lent by the discovery of the large part which micro-organisms play in the causation of disease. Surgical treatment has been revolutionised as a consequence of such knowledge, and the developments of preventive medicine, which are so striking a feature of modern practice, are largely due to the same influence. On the other hand, the methods of the physician have not been found to be so readily adapted to the modifications suggested by the facts and doctrines of bacteriology, and in some respects, indeed, the new teaching has appeared to encourage a sceptical or pessimistic attitude towards many traditional forms of medical treatment. The experience of a former day has been repeated, and just as the study of pathological anatomy compelled, it was once said, an utter disbelief in the virtues and powers of drugs, so more recently the suggestion has arisen that the social value of medical practice must be found in its ability to prevent disease rather than in the amiable delusion of its power to cure the patient.

Another mental tendency induced as a result of the appreciation of the pervading influence of micro-organisms as causes of disease is one which led to the view that such agents were in many instances sufficient in themselves to institute the diseased processes often found to be associated with their presence. Thus, for example, the tubercle bacillus was regarded as the cause of tuberculosis, not merely in the sense that without it no tuberculous disease was possible, but in the wider meaning that, given the presence of the micro-organism in the body, the disease was bound to follow. Hence the view that phthisis pulmonalis, for example, was a disease in which heredity played a large part was brushed aside as both absurd and out of date, and the bacillus was presented as the one and only responsible agent in the scheme of causation. Experience has somewhat severely corrected this teaching, and has shown that the presence of various pathogenic bacilli in the tissues is by no means the same thing as the various diseases which are attributed to them. In other words, it has to be admitted that in the production of the disease there must not only be the external agent but also a certain quality or tendency of the tissues, and that this, like other bodily qualities, may be transmitted by inheritance. From conclusions of this order there naturally arose a desire to investigate the nature of the factor contributed by the body in the development or non-development of the various specific diseases. Why, it was asked, does one individual suffer from an attack of one of these diseases, whilst another individual, placed in identical circumstances, escapes? Added to this came speculations regarding the recovery of some patients and the death of others; and also inquiries in reference to the common experience, that one attack of some of the diseases now in question bestows on the individual sufferer immunity from a second or later attack.

Here, then, are two broad positions to which opinion has been led as a consequence of the application of bacteriology to medical practice. In the one, any treatment short of the surgical removal of the diseased area, when this is possible, is regarded almost with despair. In the other, is seen some factor, contributed by the body, and playing a large share in determining both the incidence and the issue of the disease. It has now become evident that the recognition of this latter doctrine must have an important influence on questions of treatment. For it is known that the tissue agency in regard to an invasion by micro-organisms is not a passive, but an active one. Regarded at first as a vague and indefinite quality which played towards invading germs the part which a soil is popularly regarded as holding to the seed scattered over it, it has now come to be recognised as an ordered process or series of processes, which may be made the subject of precise observation and study. Further, these bodily phenomena, it is seen, are not only expressions of activity having some degree of ability to prevent, or, failing that, to limit, an attack of disease, but they are capable of being intensified on the one hand or depressed on the other; and these modifications may, without doubt, be promoted by what may fairly be called medical treatment. In this way, the transfer of attention from the invading agents or micro-organisms to the method of their reception in the tissues has conspicuously modified the doleful view in reference to treatment which the early studies of the bacteriologists undoubtedly suggested. This study of what may be called the natural resistance of the body to the causative agents of disease is likely to produce far-reaching results on treatment. It is already, even in such a disease as cancer, causing a reconsideration of surgical method and technique. Similarly, the surgeon is being urged to pause in his attempts to eradicate parts affected with tubercle, on the ground that in many such cases a natural cure will occur. Finally, the methods associated with the name of Sir A. E. Wright, whether they are or are not final in form, are based on a recognition of the possibility of assisting, directly or indirectly, those capacities of the tissues upon which a successful resistance to disease so largely depends.

